# Ab Initio Lattice Quantum Chromodynamics Calculations of Parton Physics in the Proton: Large-Momentum Effective Theory versus Short-Distance Expansion

**DOI:** 10.34133/research.0695

**Published:** 2025-05-28

**Authors:** Xiangdong Ji

**Affiliations:** Maryland Center for Fundamental Physics, Department of Physics, University of Maryland, College Park, MD 20742, USA.

## Abstract

Although equivalent in the infinite-momentum limit, large-momentum effective theory (LaMET) and short-distance operator product expansion (SDE) are 2 very different approaches to obtain parton distribution functions (PDFs) from coordinate-space correlation functions computed in a large-momentum proton through lattice quantum chromodynamics (QCD). LaMET implements a momentum-space expansion in $\Lambda_{\mathrm{QCD}}/\left[x\left(1-x\right)P^{z}\right]$ to directly calculate PDFs $f\left(x\right)$ in a middle region of Bjorken $x\in \left[x_{\min}∼\Lambda_{\mathrm{QCD}}/{\mathrm{xP}}^{z},\;x_{\max}∼1-x_{\min}\right]$. SDE applies perturbative QCD at small Euclidean distances *z* to extract a range $\left[0,\lambda_{\max}\right]$ of leading-twist correlations, $h\left(\lambda ={\mathrm{zP}}^{z}\right)$, corresponding to the Fourier transformation of PDFs. An incomplete leading-twist correlation from SDE cannot be readily converted to a momentum-space distribution, and solving its constraints on the PDFs (or the so-called “inverse problem”) involves phenomenological modeling of the missing information beyond $\lambda_{\max}$ and has no systematic control of errors. I argue that the best use of short-distance correlations is to constrain the PDFs in the LaMET-complementary regions: $x\in \left[0,x_{\min}\right]$ and $\left[x_{\max},1\right]$ through expected end-point asymptotics, and use the results of the pion valence quark distribution from the ANL/BNL collaboration to demonstrate how this can be done.

## Introduction

Parton distribution functions (PDFs) are one of the most important physical properties of the proton and neutron, and they are necessary, among others, for predicting high-energy cross sections at the large hadron collider. In the past, the best knowledge of PDFs has been gleaned from fitting the parameterizations to the data obtained from high-energy experiments over several decades [1]. In recent years, calculating parton distributions from the first principles of quantum chromodynamics (QCD) has gained considerable momentum among lattice QCD practioners. In particular, the electron-ion collider (EIC) to be built in the United States as well as a lower-energy version in China has motivated extensive studies of parton physics in the proton, including generalized parton distributions (GPDs) and transverse-momentum-dependent (TMD) PDFs. Much progress has been made in lattice calculations, and some recent summaries can be found in [2–5].

For some time, first-principles studies of PDFs are limited to their moments, which correspond to the matrix elements of local twist-2 operators [2,6,7]. Since the 1990s, methods have been proposed to directly calculate the correlation functions of operators/currents whose expansion in the large-momentum transfer *q* or Euclidean short distance *z* [short-distance expansion (SDE)] is dominated by towers of twist-2 operators, thus effectively providing a method to calculate a few lower moments at once [8–15]. The correlations with a finite range in *z*, although insufficient to determine the *x*-dependent PDFs from first principles, have also been used to phenomenologically constrain PDFs using various inverse-problem methods, including the fitting of parameterized functions as in the global analyses of PDFs [16–25].

An alternative method to calculate *x*-dependent PDFs on lattice has been proposed following Feynman’s original idea about partons [26,27], according to which they are the effective constituents of hadrons when the latter travel at the speed of light. Thus, PDFs are momentum densities of quarks and gluons in the infinite-momentum frame with the limit taken, in field theories, before the ultraviolet (UV) divergences regularized. In actual calculations, one can approximate PDFs by the momentum distributions of quarks and gluons at finite hadron momentum $P^{z}$, which are calculated with UV cutoff much larger than $P^{z}$ [28]. Different UV limits can be matched by QCD perturbation theory using effective field theory methods [29–31], because of asymptotic freedom. The resulting power corrections in ${\left(\Lambda_{\mathrm{QCD}}/P^{z}\right)}^{2}$, where $\Lambda_{\mathrm{QCD}}$ is the strong interaction scale, can in principle be systematically studied [32]. This method has been named large-momentum effective theory (LaMET), which is a general framework to calculate parton physics and light-cone correlations far beyond the scope of the collinear PDFs, including GPDs and TMD PDFs, as well as light-front wave functions [4,33].

When limited to collinear PDFs, SDE and the framework of LaMET are equivalent in the infinite-momentum limit [34]. However, this equivalence is only formal, and they are quite different at finite $P^{z}$, where the actual lattice calculations are performed [30,35]. SDE extracts the leading-twist short-range correlations in a range of light-cone distance $\left[0,\lambda_{\max}∼\left(0.2∼0.3\;\mathrm{fm}\right)P^{z}\right]$, whereas LaMET calculations yield twist-2 *x*-space PDFs in a range of momentum fraction $\begin{array}{c} x\in \left[x_{\min}∼\Lambda_{\mathrm{QCD}}/P^{z},\;x_{\max}∼1-x_{\min}\right] \end{array}$, where the kinematic limits ($\lambda_{\max}=\infty ,x_{\min}=0,x_{\max}=1$) are reached only at $P^{z}=\infty$. The short-distance correlations encode global information about PDFs, but cannot readily determine their local properties in *x*-space without models, whereas LaMET produces directly local PDFs at given *x*’s. Since high-energy experiments measure momenta of particles, the LaMET expansion is a more natural approach to calculating, rather than phenomenologically fitting, PDFs.

In this article, I discuss the contrast and complementarity of these 2 widely studied approaches to collinear PDFs. In particular, we examine how to re-use the coordinate-space data to phenomenologically determine the PDFs outside the momentum expansion region. For a given large $P^{z}$, LaMET analysis of lattice correlation data produces the most accurate information on PDFs in a range of *x*, where systematics are under control [30,36]. In the end-point regions $\left[0,x_{\min}\right]$ and $\left[1-x_{\max},1\right]$, the expansion breaks down. However, one may be able to constrain PDFs in these regions using

• The end-point behavior known from theory consideration and phenomenology. We know that, e.g., light-cone PDFs must vanish at *x* = 1. Moreover, small-x physics is constrained by Regge behavior [37] and large-x behavior by perturbative QCD power counting [38].
• Low moments of PDFs or short-distance correlations, which control the global properties of PDFs.

Of course, this information has already been used in the analysis of lattice data in the SDE approach [16–25]. However, I suggest to better use it in a different context, namely, to phenomenologically bridge the gaps in LaMET analyses. In fact, this is perhaps the most appropriate use of the phenomenological parametrization and short-distance correlation data. From the global analysis perspective, the LaMET results provide the key constraints on PDFs in the middle *x* region, which must be satisfied in any fitting.

We will begin by reviewing some of the most salient features of SDE and LaMET in the next 2 sections. We then contrast and complement the 2 approaches in SDE-Assisted PDFs in *x*-Space. Throughout the discussions, we will use the pion valence PDF calculations by the ANL/BNL group as illustration [22,36,39]. The strategy is also applicable to other PDF calculations, such as those for GPDs and distribution amplitudes (DAs), and even TMDs.

## Moments, Short-Distance Correlations, and Global Properties of Parton Distributions

Lattice QCD calculations in parton physics started from the matrix elements of local twist-2 operators, which are PDF moments [6,7], e.g.,

$$\begin{array}{c} M_{n}\left(\mu \right)=\int_{0}^{1}\left[f_{q}\left(x,\mu \right)+{\left(-1\right)}^{n}\;f_{\bar{q}}\left(x,\mu \right)\right]x^{n-1}\mathrm{dx}\; \end{array}$$ (1)

where $f_{q,\bar{q}}\left(x\right)$ are unpolarized quark and anti-quark distributions of flavor *q*. μ indicates renormalization scheme and scale, chosen usually in dimensional regularization and modified minimal subtraction ($\bar{MS}$).

Moments are meant to capture global properties of a distribution. For instance, when *n* = 1, the moment gives the area under the curve $f_{q}\left(x\right)-f_{\bar{q}}\left(x\right)$, which counts the total number of valence quarks. For PDFs, the first moments sometime provide a strong constraint on the low-x behavior, where they tend to rise due to the presence of a large number of sea quarks and gluons. In fact, when measuring the first moment of the proton’s $g_{1}\left(x\right)$ spin structure function, the small *x* contribution has a large uncertainty due to the unknown small-x behavior [40]. There is also a substantial uncertainty for the total gluon helicity ΔG from the small *x* [41].

Higher-order moments provide additional global information about PDFs. Of course, as *n* gets larger, $x^{n}$ weighs more and more toward the x∼1 region, and the moments become sensitive only to the large-x behavior. For example, if the distribution goes like ${\left(1-x\right)}^{\beta }$ near *x* = 1, the higher-order moments are strongly correlated with the value of β [22]. However, unless one knows many of them, few moments do not give us precise information about the value of $f\left(x\right)$ at a particular *x*.

To translate lower moments into local-x information about PDFs, one usually resorts to models. From general physics considerations, the PDFs vanish at *x* = 1 as ${\left(1-x\right)}^{\beta }$, and grow like $x^{\alpha }$ at small *x* [37]. This suggests a 3-parameter model for the quark distribution

$$f_{q}\left(x\right)={\mathrm{Ax}}^{\alpha }{\left(1-x\right)}^{\beta }\;$$ (2)

and similar for the antiquark. This simple model, although without physical justification in the middle-x region, has been widely used to fit parton distributions. If taking it seriously, one only needs 3 moments to get a PDF, which in fact has been quite successful in phenomenology [42,43]. A more sophisticated model would be to add a factor that is a smooth function of *x* with additional parameters [44].

Lower-order moments are closely related to the short-range behavior of the twist-2 correlation defined from PDFs,

$$h\left(\lambda ,\mu^{2}\right)=\int_{-1}^{1}{\mathrm{dxe}}^{i\lambda x}\;f\left(x,\mu^{2}\right)\;$$ (3)

Expanding the right-hand side at small λ yields,

$$h\left(\lambda ,\mu^{2}\right)=\sum_{n=0}^{\infty }M_{n+1}\left(\mu^{2}\right)\frac{{\left(i\lambda \right)}^{n}}{n!}$$ (4)

where the Taylor coefficients are just moments. Therefore, short distance or small-λ correlations are mostly determined by the lowest few moments.

**Fig. 1.**
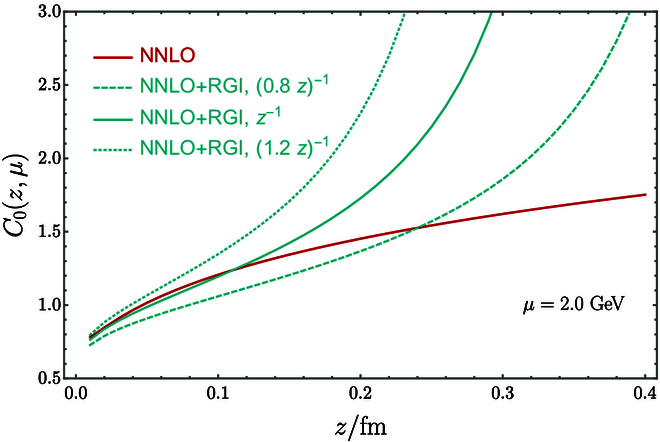
The Wilson coefficient of the number operator as a function of *z*, appearing in the quasi-PDF operator in SDE [36]. The red line is next-to-next-to leading order result. The resummed results [renormalization group improved (RGI)] are shown as solid lines, which quickly become divergent between *z* = 0.2 and *z* = 0.3 fm.

The SDE approach to calculating PDFs starts from some Euclidean correlators, e.g., $H\left(\lambda ,z^{2}\right)=\langle P^{z}|J\left(z\right)J\left(0\right)|P^{z}\rangle$, where, without loss of generality, *J* is a composite operator of quarks and gluon fields, *z* is the spatial separation along the *z* direction, and $\lambda ={\mathrm{zP}}^{z}$. At small $z^{2}\ll 1/\Lambda_{\mathrm{QCD}}^{2}$, one has the SDE or operator product expansion (OPE) [16,45,46],

$$\begin{array}{c} \begin{array}{c} H\left(\lambda ,\;z^{2}\right)=\int_{0}^{\lambda }C\left(\alpha_{s}\left(\mu \right),\;\lambda /\lambda^{\prime },{\left(\mathrm{z\mu}\right)}^{2}\right)h\left(\lambda^{\prime },\mu^{2}\right)d\lambda^{\prime }\\ +z^{2}C_{4}\left(\alpha_{s}\left(\mu \right),\lambda ,{\left(\mathrm{z\mu}\right)}^{2}\right)⊗h_{4}\left(\lambda ,\mu^{2}\right)+\ldots \end{array} \end{array}$$ (5)

where the second term on the right-hand side is a power-suppressed twist-4 contribution. *C* and $C_{4}$ are perturbation series in $\alpha_{s}$. For a reasonably large but perturbative $z^{2}$, say (0.2 fm)^2^, judging from the Wilson coefficient function of the number operator $\left(n=0\right)$ shown in Fig. 1, where the higher-twist contribution $C_{4}⊗h_{4}$ (the circled product indicates a convolution) may be dropped, one can extract the twist-2 correlation $h\left(\lambda ,\mu^{2}\right)$ up to a range about $\lambda_{\max}={\mathrm{zP}}^{z}∼3$ for *P^z^* = 3 GeV.

Thus, according to Eq. 4, instead of calculating the lowest few moments $M_{n}$, one can equivalently calculate the twist-2 correlation $h\left(\lambda ,\mu^{2}\right)$ in $\left[0,\;\lambda_{\max}\right]$ from the coordinate-space correlators. There exists no accurate estimate of the relation between $\lambda_{\max}$ and the maximal order of moment *n* to be taken into account in Eq. 4, but these 2 quantities must be roughly proportional, i.e., the larger $\lambda_{\max}$, the more moments one can extract from the correlations [14,19,22,24,47–49].

In [22], the first few moments, $\langle x^{2}\rangle$, $\langle x^{4}\rangle$, and $\langle x^{6}\rangle$, of the pion valence PDF have been extracted from fitting to the quasi-PDF correlation in the range of $z\in \left[0,\;z_{\max}∼0.8\right]$ fm. The interpretation of the large $z_{\max}$ data in OPE is only possible with fixed-order perturbative coefficient functions. The resummation of large logarithms in $\mu^{2}z^{2}$ modifies the coefficient functions drastically at large *z*, as shown in Fig. 1. Thus, in an improved analysis with next-to-leading order (NLO) resummation [39], only data up to $z_{\max}∼0.5$ fm have been used, and the determination of $\langle x^{6}\rangle$ was impossible. Moreover, if one takes into account the uncertainty in the scale setting in the resummed coefficient functions at z>0.2 fm, particularly if introducing the resummation at the next-to-next-to leading order, the error on $\langle x^{4}\rangle$ will become very larger [50]. In addition, nonperturbative contributions may contaminate the fitting for z>0.2 fm, making the moment calculation with local operators an interesting alternative [43].

Knowledge of twist-2 correlations in a finite range yields global information about PDFs, which, however, is insufficient to accurately constrain their *x*-dependence. This is similar to the quantum mechanical uncertainty principle: Only information in an infinite range in coordinate space provides precise momentum-space properties. The correlations in $\left[0,\lambda_{\max}\right]$ allow a resolution of $\Delta x∼1/\lambda_{\max}$ in momentum space. $\lambda_{\max}=3$ corresponds to a range of about 0.3 in Δx. The SDE constrains the average behavior of PDFs in this range of Δx.

Thus, any sharp construction of $f\left(x\right)$ from $h\left(\lambda \right)$ in currently accessible $\left[0,\;\lambda_{\max}\right]$ necessarily involves additional assumptions about $f\left(x\right)$ in any inverse-problem method [16], implicitly modeling the coordinate-space information beyond $\lambda_{\max}$. Using a model for $f\left(x\right)$ with few parameters is a way to correlate large and small λ, and one can fit $h\left(\lambda \right)$ in $\lambda \in \left[0,\;\lambda_{\max}\right]$ to fix the full $f\left(x\right)$. This can be viewed as a generalization to the moments-fit [42,43]. The key point, however, is that these fits will have model dependence, which is hard for error estimates, unless one has either a large number of moments or a large $\lambda_{\max}$, as in global PDF analyses with a large number of data points [1].

### How short is short?

To reduce the model uncertainty in global fits, one can try to use as large λ data as possible from lattice. With the current $P^{z}$, this means to use data at z≫0.2to0.3 fm. We found that in the literature, data even up to 0.8 fm or more have been used to make SDE analyses [16–25]. These analyses unfortunately will introduce contamination from nonperturbative contributions, which are hard to estimate and subtract (one of those is due to the ratio method of renormalization at large *z*). The important question is how small *z* must be such that one can justifiably use SDE to interpret the data.

Short-distance OPE is a double expansion, in terms of the strong coupling constant $\alpha_{s}$ and the power corrections $z\Lambda_{Q\mathrm{CD}}$. Both expansions are related. Let us consider first the $\alpha_{s}$ expansion in $\bar{MS}$ scheme mostly used in the literature.

The running of $\alpha_{s}\left(\mu \right)$ is defined perturbatively only for a limited range of momenta. The NLO beta function is definitely no longer perturbative at around μ∼0.6 GeV, where $\alpha_{s}∼0.9$. Therefore, the perturbative $\bar{MS}$ momentum scale cannot be less than about 0.6to0.7 GeV, taking into account the effects of higher-order running. Naively, this corresponds to a distance scale z∼1/μ∼0.35fm.

However, the scale μ in any perturbation series can be chosen arbitrarily. Different choices lead to different coefficients in the expansion, which compensate perfectly when a series is known to infinite order. Different choices, however, will lead to different speed of convergences. When a perturbation series is computed only to a finite order, one naturally seeks an optimal scale such that any bigger and smaller one will lead to slower convergence. This is usually dictated by the physics scales under consideration. If there is one physics scale, such as the short distance *z*, perturbation series will contain logarithms of type $\ln^{n}{\left(\mathrm{z\mu}\right)}^{2}$ (there is usually a factor of $e^{\gamma_{E}}/2$ associated with *z*, which is ∼1), which can become large if zμ strongly deviates from 1. Thus, the most natural choice of the scale is μ∼δ/z, where δ is a constant of order 1. The general practice in the perturbative QCD community is to choose δ=1/2∼2 as an estimation of error in perturbation theory.

The second expansion in OPE is in powers of the small parameter ${\left(z\Lambda_{\mathrm{QCD}}\right)}^{2}$. Usually, these contributions are smaller than the leading perturbative terms when *z* is small. However, when the perturbative expansion becomes problematic, power corrections also run out of control, which happens also around $\alpha_{s}∼1$. The power corrections are strongly influenced by the nonperturbative QCD vacuum. The SDE relies on the assumption that the propagation of quarks and gluons is given by plane waves and their perturbative scattering. However, at larger distance $z∼1/\Lambda_{\mathrm{QCD}}$, the vacuum starts to distort the plane-wave propagation strongly. Thus, an alternative criterion for the breakdown of SDE is that nonperturbative vacuum effects become important.

At a simple level, the QCD vacuum properties are described by various condensates. The most basic is the gluon condensate $\langle \left(\alpha_{s}/\pi \right)G^{2}\rangle$, which has been used in QCD sum rule calculations [51]. A recent first-principles determination of the condensate is ${\left(1.33r_{0}^{-1}\;\mathrm{GeV}\right)}^{4}={\left(0.53\;\mathrm{GeV}\right)}^{4}$ [52]. This scale indicates a breakdown of the twist expansion at distance scale $0.75r_{0}∼0.4\;\mathrm{fm}$, where the power terms have the size of the leading term, which is consistent with the estimation from the running coupling above. A well-studied model for the QCD vacuum is the instanton liquid [45], in which the classical gauge field configurations consist of various sizes of instantons generating the nonperturbative physics. One of the most important scales is the average instanton size, about 0.3 fm. Thus, the gluonic instanton configurations will have strong influences on any quark and gluon propagation at distance scale larger than 0.3 fm.

The nonperturbative vacuum effects on the quark and gluon propagators can be studied on lattice in a fixed gauge [53]. In the so-called maximally Abelian gauge where the Abelian gluons dominate the nonperturbative physics, the color confinement mechanism resembles a dual superconductor [54–56]. In recent lattice studies [57,58], it was found that the Abelian (diagonal) gluons acquire an effective mass of order 0.36 GeV, which indicates that the nonperturbative effect on the gluon propagation reaches the level of 50% at the distance scale at about 0.4 fm.

The above discussion is completely general, independent of the spatial correlators under consideration. Both QCD perturbation theory and twist-expansion hit a hard wall at around z=0.3to0.4 fm, beyond which physics become entirely nonperturbative. To stay in the perturbative region where higher-twist contributions are at the level of 10% or less, the distance scale must be smaller than 0.2 fm. In fact, if QCD physics became entirely perturbative at 0.2 fm, lattice QCD with perfect-action simulations at this lattice spacing would have produced satisfactory results, which is not the case [59].

**Fig. 2.**
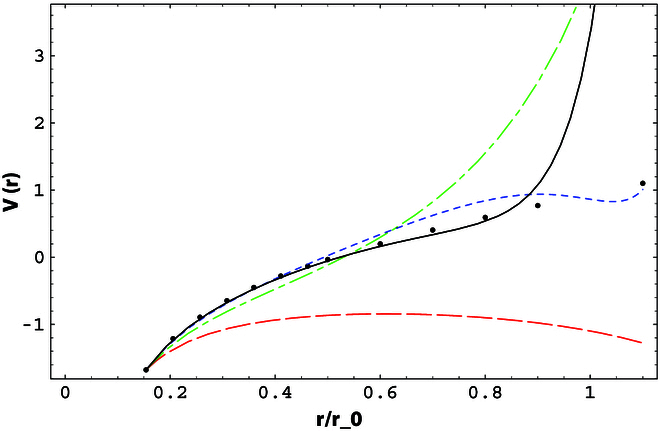
Heavy-quark potential versus *r* at tree (red dashed line), one-loop (green dash-dotted line), 2-loop (blue dotted line), and estimated 3-loop plus the renormalization group (RG) improvement for the ultrasoft logs (solid line) compared with the lattice results (dots) from [60]. The horizontal distance scale is in units of $r_{0}∼0.5$ fm.

**Fig. 3.**
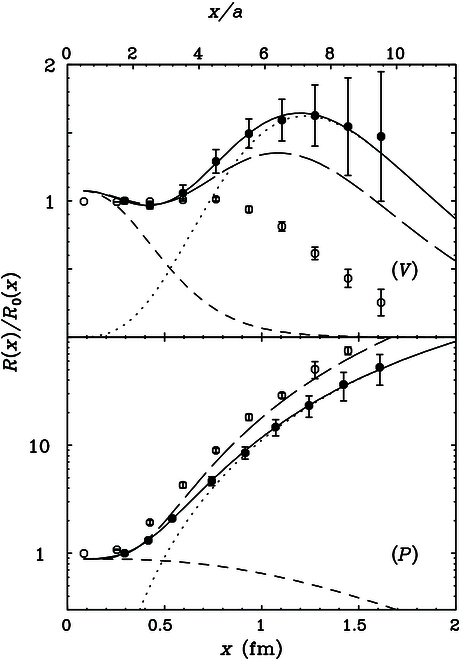
Vector and pseudo-scalar density correlation functions. The short dashed line is the perturbative contributions, and the dots are resonace contribution. Solids dots are from lattice calculations [63].

**Fig. 4.**
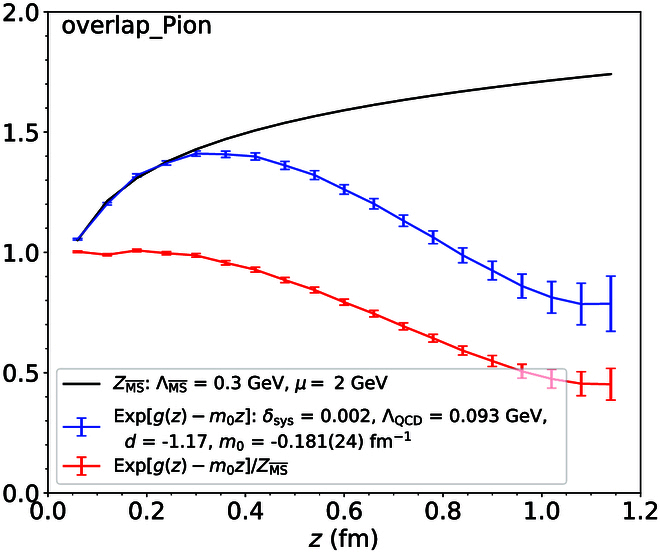
The renormalized quasi-PDF correlator in the zero-momenutm pion state (blue dost), compared with NLO perturbation theory calculations. The red dots are the ratio of the 2 [34].

**Fig. 5.**
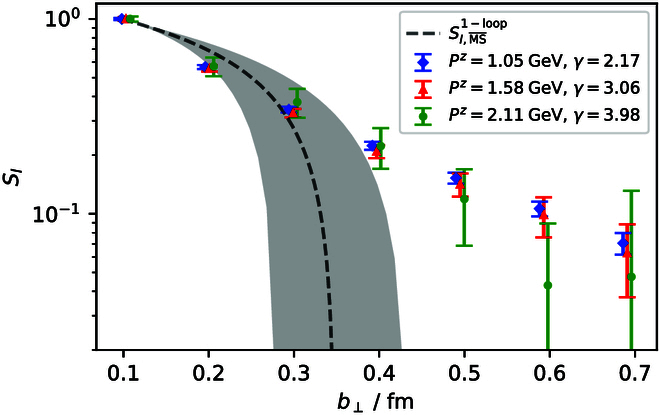
Soft function in TMD factorization. Data are from lattice, and dashed line from perturbation theory [65].

A number of case studies on spatial correlations confirm the above consideration:

• Potential between static sources [60–62]. The studies show that perturbation theory works fine up to 0.25 fm and breaks down above 0.4 fm, where nonperturbative effects set in quickly, as shown in Fig. 2.
• Hadron–hadron correlators [63]. Lattice QCD simulations have shown that the perturbative and nonperturbative contributions become about equal at around *z* = 0.5 fm. Perturbative results are fairly accurate until 0.2 fm, as shown in Fig. 3.
• Quasi-PDF correlators in zero-momentum hadron states [34]. After the careful UV renormalization, the quasi-PDF correlators are found to match to NLO perturbation theory well up to 0.25 fm, beyond which deviations occur as shown in Fig. 4.
• QCD soft function in TMD factorization [64], which is a function of transverse distance *b*: For *b* larger than 0.3 fm, the perturbative expansion breaks down [65], as shown in Fig. 5.

Thus, the perturbation series *C* and twist expansion in Eq. 5 break down at large z∼0.4 fm. Moreover, for z>0.2 fm, the correlation in $H\left(\lambda \right)$ is contaminated with nonperturbative contributions, resulting in possibly large bias of the moment analyses and global PDF fits using inverse-problem methods, which are difficult to access quantitatively [22,24,25].

## Direct calculations of PDFs in *x*-space using LaMET

LaMET aims at calculating parton densities directly at a fixed *x*, which we refer to as local information of PDFs, when the latter falls into the range where the large-momentum expansion converges.

According to Feynman, partons emerge from the infinite-momentum limit of momentum densities in hadrons. LaMET expansion begins from the quark or gluon momentum distributions $N\left(k^{z}={\mathrm{xP}}^{z},\;P^{z}\right)$ at a large but finite hadron momentum $P^{z}$ in the *z* direction, where $k^{z}$ is the longitudinal momentum of a constituent with trasverse momentum ${\vec{k}}_{⊥}$ integrated out, and takes it as a zero-order approximation (called quasi-PDF in the literature). This is not unreasonable because the difference in momentum distributions between a large and infinite momenta can naively be assessed by a dimensionless parameter $\Lambda_{\mathrm{QCD}}/P^{z}$. If $P^{z}\gg \Lambda_{\mathrm{QCD}}$, the hadron structure at this $P^{z}$ may be considered as being in the asymptotic region, where the momentum distribution shall be similar to the PDFs at infinite momentum. This can be viewed as large-momentum symmetry in analogy to heavy quark symmetry [66], in the sense that hadrons travelling at 10 GeV in energy have momentum distributions similar to those travelling at 10 TeV. In field theories, however, there are large logarithms in $P^{z}$. Feynman’s idea of momentum independence can only be realized through effective field theory [29].

Calculating the momentum density $N\left(k^{z},\;P^{z}\right)$ for gauge theories is a bit involved. One needs to start from the coordinate-space correlation

$$h\left(z,\;P^{z}\right)=\langle P^{z}\left|\psi^{†}\left(z\right)\psi \left(0\right)\right|P^{z}\rangle$$ (6)

at all −∞<z<∞, which can be difficult for large *z* where the numerical signals become noisy on a lattice. Moreover, quarks and gluons are colored particles, and gauge symmetry requires connecting the field operators by either a colored propagator or a Wilson line $W\left(z\right)$. The straight Wilson line is a preferred choice for computational simplicity. However, it leads to linear-divergent self-energy in lattice spacing *a* [67–70], which must be renormalized precisely.

The linear divergences in coordinate-space correlations $h\left(z\right)$ can be subtracted using the standard mass renormalization [34,46,71]. To reduce the discretization error near z∼0, where z→0 does not commute with a→0, a hybrid scheme has been introduced in which the short-distance correlation function at $z<z_{S}∼0.2$ fm is renormalized with a *z*-dependent lattice matrix element $Z_{o}\left(z,\;a\right)$,

$$\begin{array}{c} h^{R}\left(\lambda ,\;P^{z}/\mu_{X}\right)=\left(Z_{o}\left(z,\mu_{X}\right)/Z_{o}\left(z,\;a\right)\right)h\left(z,\;a\right)\; \end{array}$$ (7)

and $Z_{o}\left(z,\mu_{X}\right)$ is the same matrix element calculated in *X*-scheme, e.g., in $X=\bar{MS}$. For $z>z_{S}$,

$$\begin{array}{c} h^{R}\left(\lambda ,\;P^{z}/\mu_{X}\right)=C\left(\mu_{X},\;a\right)e^{\delta m\left(a\right)z}h\left(z,\;a\right) \end{array}$$ (8)

where $C\left(\mu_{X},\;a\right)$ is related perturbatively to the anomalous dimension of the heavy-light current [72] and can be determined nonperturbatively by matching to the short-distance $h^{R}$ at the boundary $z=z_{S}$.

On the other hand, it is well known that the pole mass has the so-called infrared renormalon problem in the sense that the perturbative series for the linear-divergent mass does not converge [73]. Therefore, it is important to check the renormalon consistency of the hybrid renormalization scheme. Indeed, the perturbation series $Z_{o}\left(z,\mu \right)$ has the same renormalon uncertainty as that in $\delta m\left(a\right)$ [70,74], and therefore, renormalons in both regions can be matched at $z=z_{S}$.

One can, without loss of generality, use the principal-value (PV) prescription to define the perturbation series for both $Z_{o}\left(z,\mu \right)$ and $\delta m\left(a\right)$ [75]. After subtracting the linearly divergent mass counter term, the correlation function decays exponentially at large *z* [76]

$$h^{R}\left(z,\mu \right)∼\text{exp}\left(-\bar{\Lambda }z\right)\;$$ (9)

where $\bar{\Lambda }$ is the binding energy of a light quark to a color source when the mass of the color source is calculated with a PV prescription. Studies have shown that $\bar{\Lambda }$ is on the order of few hundred MeV or so, which corresponds to the size of a typical hadron [77]. This asymptotic behavior is crucial for lattice calculations as the signal-to-noise ratio decays quickly at large *z*, and the missing information can impose significant uncertainty of Fourier transformation. Thus, the exponential decay mainly serves to reduce the uncertainties in the large-z correlation, and one can safely calculate the correlation functions up to the region around 1 to 1.5 fm to permit a physical extrapolation beyond this. The use of this physical extrapolation in LaMET is to reduce the uncertainties in the momentum density calculations of the middle-x region [36], not about predictions of PDFs at small-x where the expansion breaks down.

Thus, from the lattice data, one can obtain nonperturbative coordinate-space correlations in the $\bar{MS}$ scheme. Due to the renormalization scheme dependence, $h^{\bar{MS}}\left(\lambda ,\;P^{z}/\mu \right)$ has a discontinuity at λ = 0. This can be improved using perturbation theory [78]. The renormalized momentum distribution is just a Fourier transformation,

$$\begin{array}{c} N^{R}\left(y,\;P^{z}/\mu \right)=\int_{-\infty }^{\infty }d\lambda e^{i\lambda y}h^{R}\left(\lambda ,\;P^{z}/\mu \right) \end{array}$$ (10)

where *y* has support −∞<y<∞, which is physically sensible for a hadron with finite momentum. Using $N^{R}\left(x,\;P^{z}\right)$, one can make a large-momentum expansion for PDFs [31,32],

$$\begin{array}{c} f\left(x,\mu^{2}\right)=C_{N}\left(x,P^{z}/\mu \right)⊗N^{R}\left(x,P^{z}/\mu \right)\\ +C_{4}\left(x_{i},P^{z}/\mu \right)⊗f_{4}^{R}\left(x_{i},P^{z}\right){\left(\frac{M}{P^{z}}\right)}^{2}+\ldots \; \end{array}$$ (11)

where $C_{N}$ and $C_{4}$ are perturbation series in $\alpha_{s}\left(P^{z}\right)$, and take into account the difference between the large-momentum limits with renormalization done before hand, e.g., physical $N^{R}$ in which there is a large logarithms of $P^{z}$, and with the renormalization done afterward, which defines the standard PDFs with partons as effective theory object [29]. $C_{N}$ contains the renormalon uncertainty corresponding to the mass subtraction [70,74], which must be regulated likewise by a PV prescription. The key point of the above expression is that the infrared physics of momentum distributions in the large $P^{z}$ limit is the same as the light-cone distribution $f\left(x,\mu^{2}\right)$: Switching the limit $P^{z}\to \infty$ and a→0 does not change the soft and collinear infrared physics [29].

Power counting is key to any effective field theory [79]. Here, the small parameter is the bound state scale $\Lambda_{\mathrm{QCD}}$ or hadron masses. The obvious high-energy scale is the momentum $P^{z}$. However, careful examination indicates that the momentum of the active particle $k^{z}$ and the remnant momentum $P^{z}-k^{z}$ can also act as high-energy scales. Thus, a conservative systematic power counting is in powers of ${\left(\Lambda_{Q\mathrm{CD}}/k^{z}\right)}^{2}∼1/{\left({\mathrm{xP}}^{z}\right)}^{2}$ and ${\left(\Lambda_{Q\mathrm{CD}}/\left(P^{z}-k^{z}\right)\right)}^{2}∼1/{\left(\left(1-x\right)P^{z}\right)}^{2}$. Therefore, LaMET can only produce result in a region of $\begin{array}{c} x\in \left[x_{\min}∼\Lambda_{\mathrm{QCD}}/\left({\mathrm{xP}}^{z}\right),\right. \end{array}$$\begin{array}{c} \left.x_{\max}∼1-x_{\min}\right] \end{array}$, where the higher-order power contributions are small. The expansion is expected to break down very quickly near the end-point regions.

### How large is large?

LaMET requires large hadron momentum to work $P^{z}\gg \Lambda_{\mathrm{QCD}}$. Similar to the discussion for short-distance factorization, how large a momentum is large in LaMET? The power counting gives a more accurate answer: ${\mathrm{xP}}^{z}$ and $\left(1-x\right)P^{z}$ must be large perturbative scales, for example, 1 GeV.

A more refined result comes from the perturbative QCD matching and renormalon analysis. The one-loop matching coefficient contains the following logarithm [78]

$$\text{ln}\left(\mu^{2}/4x^{2}{\left(P^{z}\right)}^{2}\right)$$ (12)

which conjugates to $\text{ln}\mu^{2}z^{2}$ in coordinate space. Thus, it appears that $\mu =2{\mathrm{xP}}_{z}$ is the right scale setting for higher-order resummation, which correspoinds to ${\mathrm{xP}}^{z}∼0.4$ GeV. With $P^{z}$ around 2.4 GeV, the fixed-order perturbative matching shall be accurate down to x∼0.15to0.2 [50].

Note that there are soft-radiation dominating terms in the matching kernel, which goes like $\text{ln}\left(1-\xi \right)/\left(1-\xi \right)$. For *x* in the middle region, these terms do not generate large contributions due to cancellation from ξ>1 and ξ<1. However, near the end point *x* = 1, the cancellation is incomplete, and large threshold logarithms remain. These large threshold logs will effectively lower $P^{z}$ and generate an effective scale $4{\left(1-x\right)}^{2}{\left(P^{z}\right)}^{2}$. A systematic study of large-x resummation in the LaMET framework has been accomplished recently [80,81].

## SDE-Assisted PDFs in *x*-Space

The relationship between SDE and LaMET is simple in the infinite-momentum limit: They are 2 equivalent ways to define partons. However, they lead to 2 different approximation schemes for analyzing real-world data at finite hadron momenta. They are not identical expansions and will not obtain the same results from the same data, as one might have guessed naively. So which one is a better choice for which type of problem? To which degree are both approaches complementary? We try to answer these questions in this section.

### Contrast

If one’s ultimate goal is to obtain twist-2 coordinate-space correlations, corresponding to the global properties of partons, SDE is the right choice, resulting in a segment $\left[0,\lambda_{\max}\right]$ of these from the finite-momentum lattice data. On the other hand, if one is interested in *x*-space PDFs, LaMET is the the natural method to get a range $\left[x_{\min},\;x_{\max}\right]$ of distributions with controlled precision. The segments of the functions in separate coordinate and momentum spaces cannot be naively translated into each other through Fourier transformation, except in the infinite-momentum limit.

One important reason that the LaMET approach allows to obtain more precise information for *x*-space PDFs is that the momentum-space expansion utilizes the full *z*-range of data for the coordinate-space correlations and filters out their higher-twist contributions through Fourier transformation. No manual twist separation is necessary for the coordinate-space correlations even though the twist expansion itself breaks down beyond z∼0.4 fm [35]. Therefore, LaMET calculations use legitimately more large distance lattice data than SDE would allow. The correlation data at large-z are crucially important up until the exponential decay region, and the full-range correlations ensure that after Fourier transformation, the momentum distributions are dominated by twist-2 in the region $x\in \left[x_{\min},\;x_{\max}\right]$.

On the other hand, SDE can only use the correlation data up to z∼0.2 fm, beyond which one has to subtract the nonperturbative contributions, an exercise hard to control systematically. Indeed, it has been a great challenge in perturbative QCD to understand higher-twist effects quantitatively because they are intimately related to higher-order perturbation theory at the leading twist. The leading higher-twist contributions have been studied from first principles only in few cases: heavy-quark potential [60,62], heavy quark masses [77], and QCD vacuum gluon condensate [52]. Moreover, as said repeatedly, the twist expansion breaks down at z∼0.3to0.4 fm, beyond which the perturbative QCD interpretation of data is impossible.

If just using the twist-2 dominated part of the correlations from SDE, one cannot reconstruct the PDFs at any value of *x* with controlled accuracy with current lattice data, a disadvantage that cannot be overcome by any inversion methods that miss important physics information at long correlation range. A discussion of the comparison between LaMET and SDE analyses was first made in [30], and the results for the pion PDF analyses confirm the main observation of this paper [22,36].

### Complementarity

Interestingly, LaMET expansion does not exhaust all constraints on PDFs from the finite-momentum lattice data. SDE analyses can provide complementary information, which can help LaMET analyses. An important application of SDE is to study the ${\mathrm{aP}}^{z}$ artifacts of lattice data [22,24]. Here, we focus on the global constraints on parton physics from twist-2 correlations. Coupled with theoretical and phenomenological arguments that PDFs behave like $x^{\alpha }$ at small-x (Regge analysis [37]) and ${\left(1-x\right)}^{\beta }$ at large-x [82,83], short-distance correlations can be used to improve upon LaMET calculations in the end-point regions $\left[0,\;x_{\min}\right]$ and $\left[x_{\max},1\right]$.

It is straightforward to parameterize $f\left(x\right)$ in these regions in terms of a systematic expansion,

$$\begin{array}{c} f\left(x\right)=\left\{\begin{array}{c} {\mathrm{Ax}}^{\alpha }\left(1+a\sqrt{x}+\ldots \right),\;x\in \left[0,\;x_{\min}\right]\\ B{\left(1-x\right)}^{\beta }\left(1+b\left(1-x\right)+\ldots \right),x\in \left[x_{\max},1\right]\; \end{array}\right. \end{array}$$ (13)

where we have included the next-to-leading terms at small and large *x*. The inclusion of $\sqrt{x}$ is purely phenomenological, and one could try analytic terms in *x* as well [44]. One can also use more sophisticated parametrizations if well motivated or even neural network approaches as in the global analyses. The normalization *A* and *B* can be determined by continuity conditions at $x=x_{\min},x_{\max}$. More continuity conditions such as first-order derivatives can be added as additional constraints. All parameters including the exponents α and β can be fitted to either twist-2 correlations or lowest order moments, with fixed LaMET predictions on PDFs in the central-x region. This presumably will generate state-of-the-art lattice QCD PDFs with minimal bias.

**Fig. 6.**
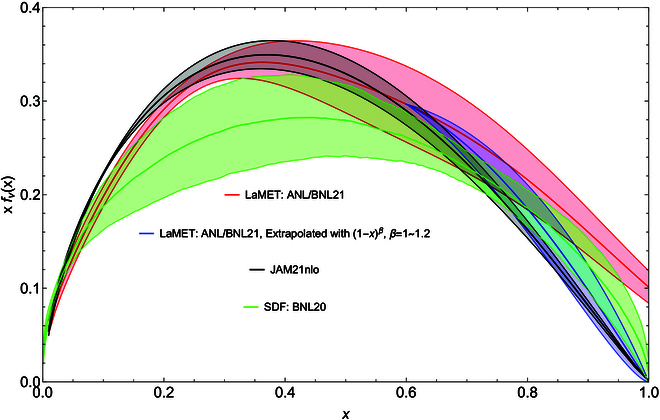
Valence pion PDFs from both LaMET (red band) and SDE (green band). The blue band is a fit to the second moment of the PDF plus LaMET result up to *x* = 0.6. The black band is the JAMnlo result from fitting to experimental data.

As an example, let us consider the pion valence distribution. The ANL/BNL group has recently produced some of the most accurate data for coordinate-space correlations in terms of quasi-PDFs [22,36]. The data were generated for pion mass $m_{\pi }∼300$ MeV at very small lattice spacings *a* = 0.06 fm and 0.04 fm. The data have been analyzed in both SDE [22] and LaMET [36], with results shown as red (LaMET) and green (SDE) bands in Fig. 6.

As one can see, the LaMET calculation generates a reasonable prediction for the valence PDF in the *x*-range roughly from 0.1 to 0.7. For x<0.1 and x>0.7, LaMET results become less reliable due to higher twists, and ultimately, the momentum-space expansion breaks down.

On the other hand, the *x*-dependent analysis of the SDE is model-dependent [22]. In particular, in the middle region, the result has a large error. This unreliability propagates to the larger-x region because of the global constraint from the short-distance correlation or lower moments. This results in a very stiff large-x distribution [22].

If one uses the LaMET result in the middle *x* region and fits the phenomenological parametrizations using the global constraints from the small-distance expansion, or the first few moments, the distribution at large-x will improve. As a quick exercise, we use $A{\left(1-x\right)}^{\beta }$ beyond $x_{\max}=0.6$ as a fitting function, and constrain β by the second moment $\langle x^{2}\rangle$, which has been calculated to high accuracy in both [22, 36] and [84]. This simple fit yields an exponent β=1.0to1.2, corresponding $\langle x^{2}\rangle =0.110$ to 0.104, shown as blue band in Fig. 6. This large-x result is more consistent with JAM (Jefferson Lab Angular Momentum) fit than the SDE fit. If one chooses $x_{\max}=0.7$, the large-x behavior will be further softened. A full fitting analysis to the entire twist-2 coordinate-space correlation including both small and large *x* regions is beyond the scope of this article. Similar complementary studies have been made for DA [85].

Finally, one might wonder how it is possible that the same data analyzed differently can generate complementary information on the PDFs. For example, why does the LaMET analysis not already exhaust all useful information? An answer follows from the first moment of PDFs. LaMET calculations are not trustable in the end-point regions and therefore cannot predict reliably the first moments. On the other hand, one can use the parton phenomenology and global properties to fix PDFs in these regions.

## Conclusion

In this paper, we have explained the similarities and differences between SDE and LaMET. In particular, we have shown how to use the twist-2 correlation functions from SDE to phenomenologically improve the LaMET predictions of PDFs in the end-point regions. We have used the recent result on the pion valence PDF as an example to demonstrate this point. Immediate applications can be made to the proton’s iso-vector PDF and meson DAs [85,86]. Furthermore, the approach discussed is entirely applicable to other parton observables, including but not limited to GPDs and TMD PDFs.

## Data Availability

There are no data generated by the author of this article.

## References

[1] Gao J, Harland-Lang L, Rojo J. Parton distributions and lattice QCD calculations: A community white paper. Phys Rep. 2018;742:1–121.

[2] Lin H-W, Nocera ER, Olness F, Orginos K, Rojo J, Accardi A, Alexandrou C, Bacchetta A, Bozzi G, Chen JW, et al. Parton distributions and lattice QCD calculations: A community white paper. Prog Part Nucl Phys. 2018;100:107–160.

[3] Cichy K, Constantinou M. A guide to light-cone PDFs from lattice QCD: An overview of approaches, techniques and results. Adv High Energy Phys. 2019;2019(1):3036904.

[4] Ji X, Liu Y-S, Liu Y, Zhang J-H, Zhao Y. Large-momentum effective theory. Rev Mod Phys. 2021;93: Article 035005.

[5] Constantinou M, Courtoy A, Ebert MA, Engelhardt M, Giani T, Hobbs T, Hou TJ, Kusina A, Kutak K, Liang J, et al. Parton distributions and lattice-QCD calculations: Toward 3D structure. Prog Part Nucl Phys. 2021;121: Article 103908.

[6] Martinelli G, Sachrajda CT. A lattice study of nucleon structure. Nucl Phys B. 1988;306(2):865.

[7] Gockeler M, Horsley R, Ilgenfritz E-M, Perlt H, Rakow PEL, Schierholz G, Schiller A. Polarized and unpolarized nucleon structure functions from lattice QCD. Phys Rev D. 1996;53:2317. 10.1103/physrevd.53.2317 10020229

[8] Aglietti U, Ciuchini M, Corbo G, Franco E, Martinelli G, Silvestrini L. Model independent determination of the light-cone wave functions for exclusive processes. Phys Lett B. 1998;441(1–2):371–375.

[9] Detmold W, Lin CJD. Deep-inelastic scattering and the operator product expansion in lattice QCD. Phys Rev D. 2006;73: Article 014501.

[10] Braun V, Müller D. Exclusive processes in position space and the pion distribution amplitude. Eur Phys J C. 2008;55:349.

[11] Radyushkin A. Quasi-parton distribution functions, momentum distributions, and pseudo-parton distribution functions. Phys Rev D. 2017;96: Article 034025.

[12] Chambers AJ, Horsley R, Nakamura Y, Perlt H, Rakow PEL, Schierholz G, Schiller A, Somfleth K, Young RD, Zanotti JM, et al. Nucleon structure functions from lattice operator product expansion. Phys Rev Lett. 2017;118: Article 242001. 28665659 10.1103/PhysRevLett.118.242001

[13] Ma Y-Q, Qiu J-W. Exploring partonic structure of hadrons using ab initio lattice QCD calculations. Phys Rev Lett. 2018;120: Article 022003. 29376680 10.1103/PhysRevLett.120.022003

[14] Bali GS, Braun VM, Gläßle B, Göckeler M, Gruber M, Hutzler F, Korcyl P, Schäfer A, Wein P, Zhang J-H. Pion distribution amplitude from Euclidean correlation functions: Exploring universality and higher-twist effects. Phys Rev D. 2018;98: Article 094507.

[15] Detmold W, Grebe AV, Kanamori I, Lin CJD, Perry RJ, Zhao Y. Parton physics from a heavy-quark operator product expansion: Formalism and Wilson coefficients. Phys Rev D. 2021;104: Article 074511.

[16] Karpie J, Orginos K, Rothkopf A, Zafeiropoulos S. Reconstructing parton distribution functions from Ioffe time data: From Bayesian methods to neural networks. J High Energy Phys. 2019;2019:57.

[17] Sufian RS, Karpie J, Egerer C, Orginos K, Qiu J-W, Richards DG. Pion valence quark distribution from matrix element calculated in lattice QCD. Phys Rev D. 2019;99: Article 074507.

[18] Joó B, Karpie J, Orginos K, Radyushkin A, Richards D, Zafeiropoulos S. Parton distribution functions from Ioffe time pseudo-distributions. J High Energy Phys. 2019;2019:81. 10.1103/PhysRevLett.125.232003 33337179

[19] Joó B, Karpie J, Orginos K, Radyushkin AV, Richards DG, Sufian RS, Zafeiropoulos S. Pion valence structure from Ioffe-time parton pseudodistribution functions. Phys Rev D. 2019;100: Article 114512.

[20] Sufian RS, Egerer C, Karpie J, Edwards RG, Joó B, Ma Y-Q, Orginos K, Qiu J-W, Richards DG. Pion valence quark distribution from current-current correlation in lattice QCD. Phys Rev D. 2020;102: Article 054508.

[21] Bhat M, Cichy K, Constantinou M, Scapellato A. Flavor nonsinglet parton distribution functions from lattice QCD at physical quark masses via the pseudodistribution approach. Phys Rev D. 2021;103: Article 034510.

[22] Gao X, Jin L, Kallidonis C, Karthik N, Mukherjee S, Petreczky P, Shugert C, Syritsyn S, Zhao Y. Valence parton distribution of the pion from lattice QCD: Approaching the continuum limit. Phys Rev D. 2020;102: Article 094513.

[23] Del Debbio L, Giani T, Karpie J, Orginos K, Radyushkin A, Zafeiropoulos S. Neural-network analysis of parton distribution functions from Ioffe-time pseudodistributions. J High Energy Phys. 2021;2021:138.

[24] Joó B, Karpie J, Orginos K, Radyushkin AV, Richards DG, Zafeiropoulos S. Parton distribution functions from Ioffe time pseudodistributions from lattice calculations: Approaching the physical point. Phys Rev Lett. 2020;125: Article 232003. 33337179 10.1103/PhysRevLett.125.232003

[25] Karpie J, Orginos K, Radyushkin A, Zafeiropoulos S. The continuum and leading twist limits of parton distribution functions in lattice QCD. J High Energy Phys. 2021;11:024.

[26] Bjorken JD, Paschos EA. Inelastic electron-proton and 𝛾-proton scattering and the structure of the nucleon. Phys Rev. 1969;185:1975.

[27] Feynman RP. Very high-energy collisions of hadrons. Phys Rev Lett. 1969;23:1415.

[28] Ji X. Parton physics on a Euclidean lattice. Phys Rev Lett. 2013;110: Article 262002. 23848864 10.1103/PhysRevLett.110.262002

[29] Ji X. Parton physics from large-momentum effective field theory. Sci China Phys Mech Astron. 2014;57:1407.

[30] Ji X. Why is LaMET an effective field theory for partonic structure? Assoc Asia Pac Phys Soc Bull. 2020;30(5):58.

[31] Ji X. Euclidean effective theory for partons in the spirit of Steven Weinberg. Nucl Phys B. 2024;1007: Article 116670.

[32] Chen J-W, Cohen SD, Ji X, Lin H-W, Zhang J-H. Nucleon helicity and transversity parton distributions from lattice QCD. Nucl Phys B. 2016;911:246.

[33] Ji X, Liu Y. Computing light-front wave functions without light-front quantization: A large-momentum effective theory approach. Phys Rev D. 2021;105: Article 076104.

[34] Huo Y-K, Su Y, Gui LC, Ji X, Li YY, Liu Y, Schäfer A, Schlemmer M, Sun P, Wang W, et al. Self-renormalization of quasi-light-front correlators on the lattice. Nucl Phys B. 2021;969: Article 115443.

[35] Ji X, Liu Y, Schäfer A, Wang W, Yang Y-B, Zhang J-H, Zhao Y. A hybrid renormalization scheme for quasi light-front correlations in large-momentum effective theory. Nucl Phys B. 2021;964: Article 115311.

[36] Gao X, Hanlon AD, Mukherjee S, Petreczky P, Scior P, Syritsyn S, Zhao Y. Lattice QCD determination of the Bjorken-𝑥 dependence of parton distribution functions at next-to-next-to-leading order. Phys Rev Lett. 2022;128: Article 142003. 35476471 10.1103/PhysRevLett.128.142003

[37] Ioffe BL, Khoze VA, Lipatov LN, *Hard processes. Vol. 1: Phenomenology, quark parton model*. Amsterdam: North-Holland; 1985.

[38] Brodsky SJ, Farrar GR. Scaling laws at large transverse momentum. Phys Rev Lett. 1973;31:1153.

[39] Gao X, Lee K, Mukherjee S, Shugert C, Zhao Y. Origin and resummation of threshold logarithms in the lattice QCD calculations of PDFs. Phys Rev D. 2021;103: Article 094504.

[40] Abe K, Akagi T, Anthony PL, Antonov R, Arnold RG, Averett T, Band HR, Bauer JM, Borel H, Bosted PE, et al. Precision measurement of the proton spin structure function g_1_^p^. Phys Rev Lett. 1995;74:346. 10058735 10.1103/PhysRevLett.74.346

[41] de Florian D, Sassot R, Stratmann M, Vogelsang W. Evidence for polarization of gluons in the proton. Phys Rev Lett. 2014;113: Article 012001. 25032920 10.1103/PhysRevLett.113.012001

[42] Detmold W, Melnitchouk W, Thomas AW. Parton distribution functions in the pion from lattice QCD. Phys Rev D. 2003;68: Article 034025.

[43] Alexandrou C, Bacchio S, Cloët I, Constantinou M, Hadjiyiannakou K, Koutsou G, Lauer C. Pion and kaon ⟨𝑥^3^⟩ from lattice QCD and PDF reconstruction from Mellin moments. Phys Rev D. 2021;104: Article 054504.

[44] Dulat S, Hou T-J, Gao J, Guzzi M, Huston J, Nadolsky P, Pumplin J, Schmidt C, Stump D, Yuan CP. New parton distribution functions from a global analysis of quantum chromodynamics. Phys Rev D. 2016;93: Article 033006.

[45] Schäfer T, Shuryak EV. Instantons in QCD. Rev Mod Phys. 1998;70(2):323.

[46] Ishikawa T, Ma Y-Q, Qiu J-W, Yoshida S. Practical quasi parton distribution functions. arXiv. 2016. 10.48550/arXiv.1609.02018

[47] Bali GS, Braun VM, Gläßle B, Göckeler M, Gruber M, Hutzler F, Korcyl P, Lang B, Schäfer A, Wein P, et al. Pion distribution amplitude from Euclidean correlation functions. Eur Phys J C. 2018;78:217.

[48] Bali GS, Braun VM, Bürger S, Göckeler M, Gruber M, Hutzler F, Korcyl P, Schäfer A, Sternbeck A, Wein P, et al. Light-cone distribution amplitudes of pseudoscalar mesons from lattice QCD. J High Energy Phys. 2019;2019:65.

[49] Detmold W, Grebe A, Kanamori I, Lin CJD, Mondal S, Perry R, Zhao Y. Parton physics from a heavy-quark operator product expansion: Lattice QCD calculation of the second moment of the pion distribution amplitude. Phys Rev D. 2021;105: Article 034506.

[50] Su Y, Holligan J, Ji X, Yao F, Zhang J-H, Zhang R. Resumming quark’s longitudinal momentum logarithms in LaMET expansion of lattice PDFs. Nucl Phys B. 2023;991: Article 116201.

[51] Shifman MA, Vainshtein AI, Zakharov VI. QCD and resonance physics. Theoretical foundations. Nucl Phys B. 1979;147(5):385.

[52] Ayala C, Lobregat X, Pineda A. Hyperasymptotic approximation to the plaquette and determination of the gluon condensate. J High Energy Phys. 2020;2020:93.

[53] Huber MQ. Nonperturbative properties of Yang-Mills theories. Phys Rep. 2020;879:1–92.

[54] Nambu Y. Strings, monopoles, and gauge fields. Phys Rev D. 1974;10(12):4262.

[55] Mandelstam S. II. Vortices and quark confinement in non-Abelian gauge theories. Phys Rep. 1976;23(3):245–249.

[56] ‘tHooft G. On the phase transition towards permanent quark confinement. Nucl Phys B. 1978;138:1–25.

[57] Gongyo S, Suganuma H. Two-dimensional phase structure of SU(2) gauge-Higgs model. Phys Rev D. 2013;87: Article 074506.

[58] Schröck M, Vogt H. Cauchy-horizon singularity inside perturbed Kerr black holes. Phys Rev D. 2016;93: Article 014501.

[59] DeGrand TA, Hasenfratz A, Hasenfratz P, Niedermayer F, Wiese U. Towards a perfect fixed point action for SU(3) gauge theory. Nucl Phys B Proc Suppl. 1995;42:67–72.

[60] Pineda A. The static potential: Lattice versus perturbation theory in a renormalon-based approach. J Phys G. 2003;29(2):371.

[61] A. Bazavov, N. Brambilla, I. Tormo, Garcia X, Petreczky P, Soto J, Vairo A, Determination of alpha_s from the QCD static energy: An update. *Phys Rev D*. 2014;90:074038.

[62] Ayala C, Lobregat X, Pineda A. Determination of α(*M_Z_*) from an hyperasymptotic approximation to the energy of a static quark-antiquark pair. J High Energy Phys. 2020;2020:16.

[63] Chu MC, Grandy JM, Huang S, Negele JW. Correlation functions of hadron currents in the QCD vacuum calculated in lattice QCD. Phys Rev D. 1993;48(7):3340. 10.1103/physrevd.48.3340 10016592

[64] Collins JC, Soper DE. Parton distribution and decay functions. Nucl Phys B. 1982;197(3):446–447.

[65] Zhang Q-A, Hua J, Huo Y, Ji X, Liu Y, Liu YS, Schlemmer M, Schäfer A, Sun P, Wang W, et al. Lattice-QCD calculations of TMD soft function through large-momentum effective theory. Phys Rev Lett. 2020;125(19): Article 192001. 33216591 10.1103/PhysRevLett.125.192001

[66] Georgi H. An effective field theory for heavy quarks at low energies. Phys Lett B. 1990;240(3–4):447.

[67] Ji X, Zhang J-H, Zhao Y. Back-to-back jets: Fourier transform from *b* to k_T_. Phys Rev Lett. 2018;120: Article 112001. 29601738 10.1103/PhysRevLett.120.112001

[68] Green J, Jansen K, Steffens F. Nonperturbative renormalization of nonlocal quark bilinears for parton quasidistribution functions on the lattice using an auxiliary field. Phys Rev Lett. 2018;121(2): Article 022004. 30085753 10.1103/PhysRevLett.121.022004

[69] Ishikawa T, Ma Y-Q, Qiu J-W, Yoshida S. Renormalizability of quasiparton distribution functions. Phys Rev D. 2017;96: Article 094019.

[70] Zhang R, Holligan J, Ji X, Su Y. Leading power accuracy in lattice calculations of parton distributions. Phys Lett B. 2023;844: Article 138081.

[71] Chen J-W, Ji X, Zhang J-H. Improved quasi parton distribution through Wilson line renormalization. Nucl Phys B. 2017;915, 1–919.

[72] Ji X-D, Musolf MJ. Sub-leading logarithmic mass-dependence in heavy meson form factors. Phys Lett B. 1991;257(3–4):409–413.

[73] Hooft G. Can we make sense out of quantum chromodynamics? Subnucl Ser. 1979;15:943.

[74] Braun VM, Vladimirov A, Zhang J-H. Power corrections and renormalons in parton quasidistributions. Phys Rev D. 2019;99(1): Article 014013.

[75] Ayala C, Lobregat X, Pineda A. Superasymptotic and hyperasymptotic approximation to the operator product expansion. Phys Rev D. 2019;99(7): Article 074019.

[76] Burkardt M, Grandy JM, Negele JW. Calculation and interpretation of hadron correlation functions in lattice QCD. Ann Phys. 1995;238(2):441–472.

[77] Ayala C, Lobregat X, Pineda A. Hyperasymptotic approximation to the top, bottom, and charm pole mass. Phys Rev D. 2020;101(3): Article 034002.

[78] Izubuchi T, Ji X, Jin L, Stewart IW, Zhao Y. Factorization theorem relating Euclidean and light-cone parton distributions. Phys Rev D. 2018;98: Article 056004.

[79] Weinberg S. On the development of effective field theory. Eur Phys J H. 2021;46:6.

[80] Ji X, Liu Y, Su Y. Threshold resummation for computing large-*x* parton distribution through large-momentum effective theory. J High Energy Phys. 2023;2023:37.

[81] Ji X, Liu Y, Su Y, Zhang R. Effects of threshold resummation for large-x PDF in large momentum effective theory. J High Energy Phys. 2025;2025:45.

[82] Drell SD, Yan T-M. Connection of elastic electromagnetic nucleon form factors at large 𝑄^2^ and deep inelastic structure functions near threshold. Phys Rev Lett. 1970;24(4):181.

[83] West GB. Phenomenological model for the electromagnetic structure of the proton. Phys Rev Lett. 1970;24(21):1206.

[84] Alexandrou C, Bacchio S, Cloet I, Constantinou M, Hadjiyiannakou K, Koutsou G, Lauer C. Mellin moments ⟨𝑥⟩ and ⟨𝑥^2^⟩ for the pion and kaon from lattice QCD. Phys Rev D. 2021;103(1): Article 014508.

[85] Holligan J, Ji X, Lin H-W, Su Y, Zhang R. Precision control in lattice calculation of x-dependent pion distribution amplitude. Nucl Phys B. 2023;993: Article 116282.

[86] Hua J, Chu M-H, He F-C, He J-C, Ji X, Schafer A, Su Y, Sun P, Wang W, Xu J, et al. Pion and kaon distribution amplitudes from lattice QCD. Phys Rev Lett. 2022;129(13): Article 132001. 36206420 10.1103/PhysRevLett.129.132001

